# Metastatic Endometrioid Carcinoma Mimicking a Subungual Melanoma

**DOI:** 10.3390/ijerph192114494

**Published:** 2022-11-04

**Authors:** Jena C. Glavy, Shian L. Peterson, Jonathan Strain, Kevin Byrd, James H. Flint

**Affiliations:** 1 Department of OBGYN, Navy Medicine Readiness and Training Command, San Diego, CA 92134, USA; 2 Department of Orthopedic Surgery, Navy Medicine Readiness and Training Command, San Diego, CA 92134, USA; 3 Department of Pathology, Navy Medicine Readiness and Training Command, San Diego, CA 92134, USA

**Keywords:** endometrial adenocarcinoma, bone metastasis, subungual, acral metastasis, exophytic

## Abstract

Case: We report a case of a 76-year-old female with a stage IB, grade I endometrioid endometrial carcinoma who presented with right-hip pain and an enlarging black, exophytic, subungual lesion on her right-small-finger distal phalanx. Clinically, the distal phalanx lesion was suspicious for a subungual melanoma; however, advanced imaging suggested metastatic disease, with lesions in the acetabulum, lungs, brain, vulva, and vagina. Conclusion: Partial amputation of the right, small finger and vulvar biopsies confirmed an endometrial carcinoma. To our knowledge, this is the first described case of endometrial adenocarcinoma metastasis to the phalanx of an upper extremity, mimicking a subungual melanoma.

## 1. Introduction

An endometrial carcinoma is the most common malignancy in developed countries and the fourth most common cancer in women in the US [1,2]. The mainstay of treatment in early-stage disease is surgical staging with total hysterectomy, bilateral salpingectomy, lymph node evaluation, and evaluation for extrauterine disease. Most women have a favorable prognosis, as most cases are confined to the uterus, with an 89.6% and 77.6% 5-year survival rate for stages IA and IB [3].

In stage I and II diseases, the recurrence rates are 4.8% and 17.6%, respectively [4]. Distant metastases are rare in grade 1 tumors, with lymph node involvement in 3.3%, pelvic metastasis in 4.6%, and distant metastasis in 2.4% [5]. Most recurrences occur in the first three years after treatment, and typical metastatic sites include vagina, pelvic and para-aortic lymph nodes, peritoneum, and lungs. Atypical sites of recurrence involve abdominal organs, bones, the brain, and muscle. Women with recurrence confined to the vagina have a better prognosis compared to pelvic and distant recurrences [6]. Bone dissemination is uncommon and thought to occur in <1% of cases [7]. Little information on osseous metastasis is available in the medical literature.

## 2. Case

**Figure 1:**
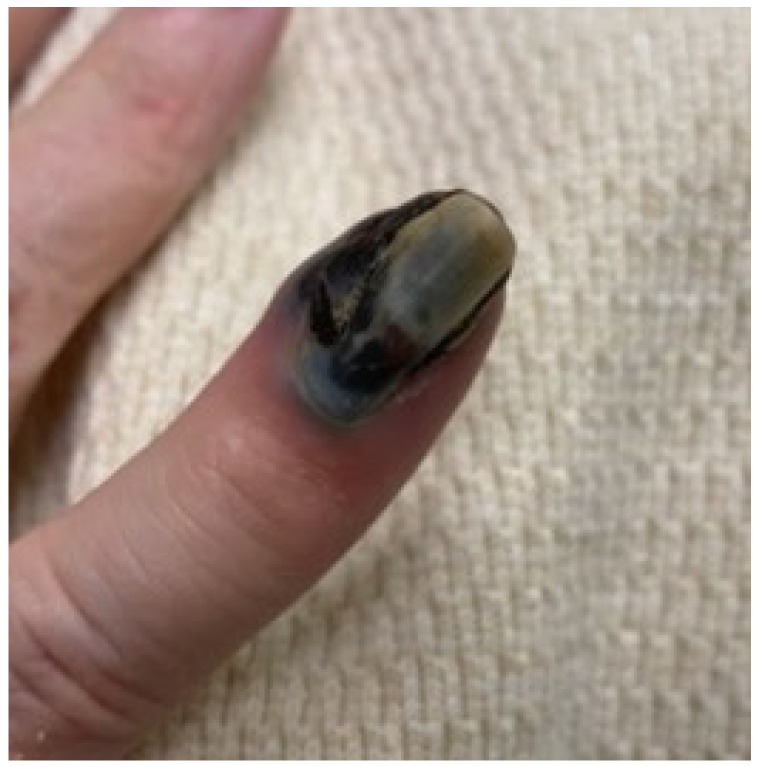
Presenting image of the acral metastasis, with a pathologic assessment consistent with a metastatic endometrial adenocarcinoma.

We present a 76-year-old nullipara postmenopausal female with a history of stage 1B, grade 1 endometrioid endometrial adenocarcinoma status post-robot-assisted total laparoscopic hysterectomy, bilateral salpingectomy, and pelvic lymph node dissection 10 months earlier. The initial gynecologic specimen at the time of surgery was noted to have a depth of invasion of 63%, without lymphovascular invasion or lower uterine segment involvement. Left-pelvic sentinel lymph node sampling and six right pelvic nodes were all negative for malignancy. She presented with a rapidly expansile, painful, fungating subungual mass on her right-small-finger distal phalanx, which had the classic appearance of a subungual melanoma (Figure 1), given its color and location. She also reported concomitant right-hip pain and a vulvar mass. Her symptoms developed over a 10-day period. The widely metastatic presentation was more concerning for melanomas, and recurrent endometrial cancer was felt to be less likely as this pattern of metastatic disease would be remarkably rare.

**Figure 2:**
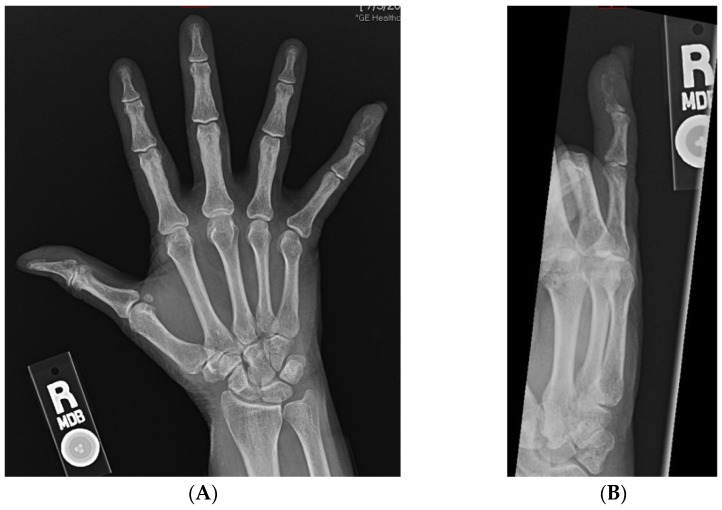
(**A**,**B**): AP and oblique radiographs of the right hand demonstrating lytic destruction and pathologic fracture of the distal phalanx of the small finger.

**Figure 3:**
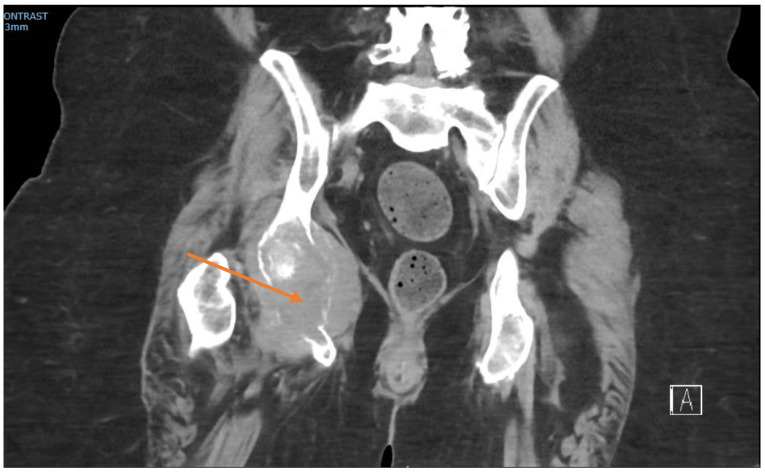
Coronal CT pelvis showing lytic destruction of the right ischium (arrow), with soft-tissue mass extension.

At the time of presentation, radiographs of the right hand showed a destructive lesion in the distal phalanx of the small finger, with a pathologic fracture and an associated soft-tissue mass (Figure 2). An MRI of the right hand showed a 1.5 cm destructive lesion within the small-finger distal phalanx, with subungual soft-tissue extension. Upon a further work-up, CTs of the abdomen/pelvis demonstrated a lytic lesion of the right acetabulum extending into the ischium (Figure 3). An MRI of the pelvis showed additional enhancing masses at the anterior right vagina and anterior vulva. Further advanced imaging revealed multiple, bilateral pulmonary nodules on a CT of the chest and brain metastases. PET/CT was consistent, and further visualized a left external iliac lymph node and nonspecific focal uptake in the right forefoot.

Given the extent of bone destruction, clinical presentation, and concern for melanoma, the patient was taken to the operating room for excisional biopsy and tissue diagnosis via a partial amputation of her right small finger. A fish-mouth incision over the middle phalanx of the right, small finger was created, just proximal to the lesion. The digital neurovascular structures were identified and transected. Care was taken to identify the flexor digitorum profundus, and it was sharply transected. The terminal extensor tendon was similarly identified and transected. A sagittal saw was then used to create a transverse bone cut in the middle phalanx, and the specimen was sent to pathology. The remaining tissue was healthy. Meticulous hemostasis was achieved, and the incision closed. Negative margins were noted on the specimen’s pathology.

**Figure 4:**
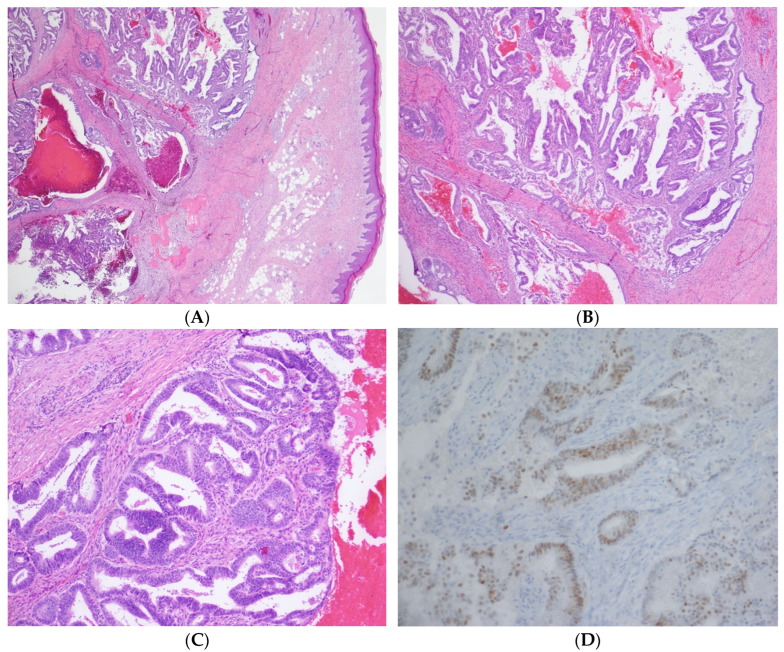
(**A**) 20× magnification: The image shows a subcutaneous mass consisting of endometrial glands and stroma in and around bone. (**B**) 40× magnification: The tumor has endometrial glands with complex architecture, including papillary and cribriform growth patterns. (**C**) 100× magnification: At higher power, the glands have pseudostratified columnar cells with round nuclei. Note the hemorrhage and endometrial stroma. (**D**) 200× magnification: The cells are positive with PAX8 immunohistochemical stain, consistent with the known endometrial adenocarcinoma primary.

Gynecologic oncology performed a concurrent gynecologic exam under anesthesia and biopsies of the concerning lesions. All biopsies indicated adenocarcinomas, with a glandular formation, and PAX-8 positivity on immunohistochemistry (Figure 4). The finger lesion showed abundant hemorrhages, including areas with hemorrhages extending into the epidermis. The location in conjunction with the hemorrhage contributed to the clinical impression of a pigmented lesion, such as a melanoma. The patient was diagnosed with a metastatic recurrence of endometrial adenocarcinoma, with metastases to the right acetabulum, pulmonary nodules, and brain lesions. She was started on Megestrol acetate for hormonal therapy for her metastatic disease.

She was referred to radiation oncology for a discussion of palliative radiation to her brain and right ischium, and a plan for check-point-inhibitor therapy, systemic therapy, and osteoclast inhibitor therapy. The patient completed 2 cycles of pembrolizumab and whole-brain radiation. Unfortunately, she succumbed to disease 2 months later, presumed to be secondary to rapidly progressive disease. Autopsy was declined.

## 3. Discussion

Bone metastases of endometrial cancer are rare, with few case reports in the literature. When osseous metastasis does occur, it usually presents in advanced stage disease and portends poor overall survival and high mortality rates [7,8,9,10].

Grade 1 endometrial cancers are typically thought of as low risk; however, they can be accompanied by high-risk features. In one study of 3867 patients with grade 1 disease, 84.5% were classified as stage I. However, approximately 10% of grade 1 cancers have deep myometrial involvement. Younger age and earlier stage are important prognostic factors in grade 1 disease [5].

Our patient had one high-risk feature. The tumor was histologically defined as grade 1 and surgically staged as IB with a depth of invasion at 63%. This places her on the border of the high-intermediate-risk subgroup for age > 70 years and outer 1/3 myometrial invasion [11]. While vaginal brachytherapy was recommended, treatment was delayed due to COVID-19 quarantine and patient concerns regarding the avoidance of healthcare settings.

Her Initial presenting symptoms were pain in the right hip and a rapidly growing black, fungating mass on her right small finger that enveloped the distal phalanx and uprooted the nailbed. The clinical appearance was typical of an exophytic, subungual melanoma. With imaging showing bone, lung, and brain metastases, her pattern of disease was highly suspicious for a metastatic melanoma. However, no features of melanoma were present on the histology. No published reports found in our search describe metastases to the phalanges of the finger from endometrial cancer.

Pain is a common presenting symptom, with the majority of cases presenting for evaluation due to new bony pain [12]. Thus, in patients who report new pain in the setting of known endometrial cancer, regardless of stage and grade, considerations should be made to evaluate for the presence of metastases with advanced imaging, such as CT, MRI, and PET/CT, as these modalities have a high detection rate.

Bone metastases are thought to result from hematologic distribution as a reflection of widespread metastatic disease. The exact mechanism is not fully understood and could result from the spread into Batson’s plexus, possibly originating from local lymphatic invasion [13]. The presence of deep myometrial invasion was the strongest predictor of hematogenous dissemination [14]. A handful of literature reviews have been published over the last 10 years. Smaller retrospective studies have reported the rate of bone dissemination at 0.4–3.1% [7,8,9,15].

In a review of 19 patients, bone metastases were found to most commonly occur in the axial skeleton, especially the spine (44.8%) and hip (13.8%) [7]. The pelvis is the most usual location, followed by the spine [5]. Acral metastasis is very rare, making up 0.1% of all bone metastasis, and is associated with a poor prognosis [16]. In a subungual melanoma of the hand, distal amputation appears to confer survival benefits; however, there is limited literature to guide definitive treatment [17].

The diagnosis of osseous metastasis confers a poor prognosis. The median survival rate for bone metastases in endometrial cancer has been reported as 6–15 months in earlier retrospective reviews [7,8,9,15]. Isolated bone metastases appear to be associated with better outcomes. Performance status was an independent prognostic factor. Additionally, type II endometrial carcinoma has a shorter life expectancy than type I. Most patients with bone metastases have moderate or poorly differentiated tumors [7,8,10].

After a complete staging work-up, we treated our patient with a partial amputation of the right, small finger to achieve local control and obtain diagnostic tissue, and to improve function and alleviate pain. She was also treated with hormonal therapy, osteoclast-inhibitor therapy (for bone metastasis), and whole-brain radiation. She also received two cycles of pembrolizumab, as her tumor was microsatellite instability-high and this treatment would allow concurrent radiation therapy. The optimal management of osseous dissemination is unknown. Little data exist regarding the management of bone metastasis in endometrial cancer, and even less literature exists with respect to acral metastasis. Uccella et al. [7] demonstrated a suggestion of longer survival rates with a multimodal approach at 33 months versus radiotherapy alone at 20 months; however, this was not statistically significant.

Treatment should be tailored to the individual patient, and an effort to identify other sites of metastatic disease should be meticulously conducted. Treatment regimens consider the patient’s performance status, extent of symptoms, location of bony metastases, number of extraosseous metastases, and previous treatment. The reported strategies at present include surgical treatment of metastatic lesions, radiation therapy, chemotherapy, and hormone therapy.

Radiation therapy directed to the bony lesion can be used as a palliative treatment, but can also be effective for pain relief and mitigate progression, especially in single-bone metastasis [18]. When there is widespread disease, systemic chemotherapy can be considered [19]. Amputation may be beneficial in managing painful metastases to the fingers and toes and increase patient comfort [20]. Bisphosphonates may also offer therapeutic values, as extrapolated data from bone metastasis from breast cancer show a reduced risk of skeletal-related events and decreased bone pain [21,22].

## 4. Conclusions

This paper described a rare acral metastasis from endometrial cancer, mimicking a malignant melanoma. Although infrequent, low-grade, early stage cancers have the potential to be aggressively metastatic, as highlighted in this case, patients with osseous metastases have poor outcomes with no standard therapy in this scenario. We emphasized the importance of a swift, but thorough, assessment and treatment of subungual lesions and the need for a multi-disciplinary approach to patient care. Further research is needed to investigate treatment strategies for bone metastasis in endometrial cancer.

## Data Availability

Data sharing is not applicable to this article as no new data were created or analyzed in this study.
